# Supplementary material for: Patterns of Intron Gain and Loss in Fungi
Source: PLoS Biol. 2004 Nov 30;2(12):e422. doi: 10.1371/journal.pbio.0020422 (PMC532390; doi:10.1371/journal.pbio.0020422)
Supplement: Table S1 — Also available at http://genes.mit.edu/NielsenEtAl/. (4.3 MB ZIP). [file pbio.0020422.st001.zip › NielsenEtAl/html/1379.html]

AN2119.1.NCU04090.1.MG00819.1.FG09254.1


```
 CLUSTAL W (1.82) Multiple Sequence Alignments - Introns Inserted


Sequence 1: NCU04090.1	1162 aa
Sequence 2: MG00819.1	1275 aa
Sequence 3: FG09254.1	1295 aa
Sequence 4: AN2119.1	1271 aa
Alignment Length: 1309 aa
Number Identitical Residues: 563 aa
Alignment Score (without introns) 30183


MG00819.1 	-----~------------------MARISSLALSASLLLASLAAAEQPWTQKHEAGRCAM
NCU04090.1	-----~------------------------------------------------------
FG09254.1 	MTQPE0INDSIPIHRRVRPNHLEMHHSIVSQLGAAAVLFAGLGFAEQ-YTPKHEPGRCAF
AN2119.1  	-----~------------------MRGLRFIPIVAALSFPAHSFAQG-KTRIHENGRCAI
          	                                  ::   .. . :.   :   . . .: 

MG00819.1 	RGHCGKKSWFGKELPCVDNGPAENPDEDFRKLLVDLCGPKWETGPVCCDKSQ0VESLKSE
NCU04090.1	----------------------------------------------------~-------
FG09254.1 	RGHCGKQSFFGKELPCVDNGLAEDPEEELRNELVGLCGEKWRTGPVCCTLDQ~VKSLKSE
AN2119.1  	RGHCGKQSFFGGELPCPDNDAAREPEAAVREKLVNLCGAKWQEGPVCCEEEQ0IDALSKN
          	 . ....:  . . .. ... : ...    .   . .. .   .. ..  ..  .: ...

MG00819.1 	LSTPRQIVSSCPACKDNFYNLFCTFTCSPDQSLFVNVTKAQEKN-GKLQITELDQLISSE
NCU04090.1	--------------------MFCTFTCSPNQSLFVNVTKTIEKK-GKELVTELDQLISEE
FG09254.1 	LGTPNTLIGSCPACKDNFFNLFCTFTCSPDQSTFINVTDSAPKN-GKNLVTELDHLVSEK
AN2119.1  	LKLAEGIISSCPACKDNFFNIFCTFTCSPDQSLFINVTKTEKGNSGKELVTELDNIWSEE
          	   .    .:..:....  .:********:** *:***.:   ::**  :****:: *.:

MG00819.1 	YGTGFYDSCKDVKFGPSNSKAMDFIGGGAKNYTQLLKFLGDEK-AIGSPFQINFPTEYSE
NCU04090.1	YGTGFYNSCKDVKFGPTNSRAMDLIGGGAKNYTQLLKFLGQER-FGGSPFQINFPVEYAE
FG09254.1 	YGSGFYDSCKEVKFGGANSRAMDLIGGGAKNYTEMLTFLGNKKPFAGSPFQINFPTQQKV
AN2119.1  	YQSGFYDSCKNVKNGASGGKAIDFIGGGAKDYQQFLKFLGDKK-FLGSPFQINYHTEPPE
          	* :***:***:** * :..:*:*:******:* ::*.***:::   *******: .:   

MG00819.1 	P--AMSPREMKPKRCNDDDPNFRCACVDCPQVCPELPAVKESGSCHVGSLPCLSFAAIFT
NCU04090.1	P--DMKPLPMKPKKCNDEDPNFRCACVDCPEICPTLPDVEQAGSCHVGALPCLSFASILT
FG09254.1 	P--KLQPVDMKPKKCNDEDPNYRCVCVDCPEVCAKLPEVKDSKSCKVGLLPCLSFASIFV
AN2119.1  	DSQGMQALPIHPKACNDADPAYRCSCVDCPDVCPELPAIKTEEHCHVGLLPCLSFSVILI
          	 :. :..  ::** *** ** :** *****::*. ** ::    *:** ******: *: 

MG00819.1 	YSIILFSLAVALTGGFVLKKHNERRRERLRLLQDPVRSDDEDEGDLVHNNAMLDRPQSNY
NCU04090.1	YSVILFISIAAVVGHVAWKRHAKRRSERLRLLTDAAPSDDEDEGDLTQNVAMIDRPQKTY
FG09254.1 	YGVLLSTLILAVTGHIAYQKYSQHRVERTRLLHESSHSDDEDEGGPVDTEAMRERPTKRY
AN2119.1  	YSVFLLG-VAGFSSYFTYRERRYRKPERVRLLQDPNPSDDEDEGDIVHAGGHLEYPHGYY
          	*.::*     .. . .. :.   :: ** *** :.  *******. ..  .  : *   *

MG00819.1 	PVNSWCDSAFSKLGHTAARFPGITIISSLIIVAVLSLGLFRFDIEKDPARLWVSPTSAAA
NCU04090.1	IINTWCDSAFSKLGYVAATFPAITIVTSILIASILSLGWFHFELEKNPARLWVSPTSPAA
FG09254.1 	WVNDRCDRGFYQLGHIAARFPGWCIGLSLLFVGILSIGLFRFDLEKEPARLWVSPSSAAA
AN2119.1  	KLNSMLDTVFSRIGSVCARFPALTIISSVVAVVLLSLGWLRFAVETDPVRLWVSPTSAAA
          	 :*   *  * ::*  .* **.  *  *:: . :**:* ::* :*.:*.******:*.**

MG00819.1 	QEKAFFDANFGPFYRAEKIFLVNDTNPSSPGPVLSYDNLIWWIDVENSVKQLKGPRFGAM
NCU04090.1	EEKAFFDSHFGAFYRAEKVFLVNDTQPSGPGPVLSRDTLLWWMDVEKSVAALKGSNYGSS
FG09254.1 	QEKAYFDENFGPFYRAEKIFLANDTNPSGPGPVLSYDTLKWWIEVEESVKKIESPVYGKY
AN2119.1  	KEKAFFDENFGPFYRAEQAFLVNDDE-TGDGRVLDYDTLTWWFGVESRIRRVISLDRGLS
          	:***:** :**.*****: **.** : :. * **. *.* **: **. :  : .   *  

MG00819.1 	LQDVCLKPTGSACVVQSVAAYFGNDADNVSKGGWKGNLRDCARSP--VECRPDFGQPIDP
NCU04090.1	FQDLCLKPTGDACVVQSVAAYFQDDPDSVDPETWQSTLRTCAASP--VECRPAYGQPLDP
FG09254.1 	FQDLCFKPSNDACVVQSVSAYWHSK-GGLDPQTWKDDIRACAKSP--VDCRPDFGQPIEP
AN2119.1  	LDDICYKPTGDACVIQSVTGYFGGSLSNLDPDTWQDRLTHCASSPGDASCLPDFSQPLRP
          	::*:* **:..***:***:.*: .. ..:.   *:. :  ** **....* * :.**: *

MG00819.1 	GMILGGYGAGDDIADAQAMTVTWVLNNFPEGTSEEARAMDFEEALKNRLLKLQEEAADRG
NCU04090.1	SMILGGYPEGGNVAEASAMTVTWVLINPSENSPEVDRAMDWEVALKNRLLEVQDEAKERG
FG09254.1 	NMIFGGYG--DDIVDAHAITVTWVVNNAKEGTDAIARAVDWETALRDRLLEVQEEAKERG
AN2119.1  	EMILGGYEDSGNVLDAKALIVTWVVNNHAPGSEEEAEAIDWEDTFRGIFQVVQEEAKNRG
          	 **:***  ..:: :* *: ****: *   .:    .*:*:* :::. :  :*:** :**

MG00819.1 	LRLSFSTEISLEQELNKSTNTDAKIIVISYIVMFLYASIALGSTTLNFREFFRNKSLFFV
NCU04090.1	LRLSFSTEISLEEELNKSTNTDAKIIVISYIIMFLYASLALGSTTLTFKDLIRNPAVSLV
FG09254.1 	LRLSFNTEISLEQELNKSTNTDAKIIVISYIVMFVYACMALGTP---LKHIFRNPAVLLV
AN2119.1  	LRVSFTTEASVEQELNKSSNTDAKIVVISYIIMFIYASLALGSVTMTWRSLINNPANALV
          	**:**.** *:*:*****:******:*****:**:**.:***: : . : ::.* :  :*

MG00819.1 	QSKFGLGIVGIAIVLLSIMASIGLFSWFGLKVTLIIVDVIPFIVLAVGVDNIFLIVHEFE
NCU04090.1	ESKFTLGIVGIVIVLMSITASIGLFSWAGLRATLIIVDVIPFIVLAVGVDNIFLIVHEFE
FG09254.1 	ESKVTLGLVGIIIVLMSIAASIGFFSWVGLKATLIIVEVIPFIVLAVGVDNIFLIVHELE
AN2119.1  	QSKFTLGVVGIVIVLMSVSASVGLFSAAGVKVTLIIAEVIPFLVLAVGVDNIFLIVYEFE
          	:**. **:*** ***:*: **:*:**  *::.****.:****:*************:*:*

MG00819.1 	RVNISHPDLDVELRIAKALGRMGPSILFSAVTETASFALGAFVGMPAVRNFAIYAAGAVF
NCU04090.1	RVNVSYPDDMVEARISRALGRMGPSILFSALTETASFALGAFVGMPAVRNFAIYAAGAVF
FG09254.1 	RVNTSFPDQMVEERVARALGRMGPSILFSALTETVAFALGTAVGMPAVRNFAAYAAGAVF
AN2119.1  	RLNVSHPDEEIDERISRAIGRIGPSIFLSAITETVAFALGVFVGMPAVRNFAIYAAGAVF
          	*:* *.**  :: *:::*:**:****::**:***.:****. ********** *******

MG00819.1 	INALLQVTMFVSFLTLNQQRVEDCRMDLFPCVQLKSARIHLNG-TGN--LGPRYHEAPQE
NCU04090.1	INAILQVTMFVSVLTLNQIRVEDSRADCFPCIQIKSARVHLAS-NGAG-PAPVYLEAPEE
FG09254.1 	VNAVLQMTMFVSFLSLNQMRVEDHRCELWPWWQITKARIHLNGSNGFAQGGGRGSDMAEE
AN2119.1  	INAVLQITMFVSVLALNQKRVESLRADCIPCLTVRKAHSGMPEDLAFD-------DQDRE
          	:**:**:*****.*:*** ***. * :  *   : .*:  :  . .         :  .*

MG00819.1 	SMLQQFIRKYYAPALLGKKVKAVVVLVFLGVFTAGVSLIPEVELGLDQRVAIPDDSYLIP
NCU04090.1	SYLQQFIRKVYAPRLLGKKTKAVIVMIFLGVFAAGVALIPEVKLGLDQRVAIPDDSYLIP
FG09254.1 	SLLQVFIKNTYAPRLLGKKVKLAVVTIFLGMFAGGLALLPKIQLGLDQRVAIPDGSYLIP
AN2119.1  	GILQKFIRKVYAPLLLNRRVKVVVVITFLGILAAGLALTPEVAMGLDQRIALPSDSYLID
          	. ** **:: *** **.::.* .:*  ***:::.*::* *:: :*****:*:*..**** 

MG00819.1 	YFNDLYAYFESGPPVYFVTKESNFTQREHQQEVCARFTTCNELSMTNILEQERKRPEISY
NCU04090.1	YFNDLYEYLNTGPPVYFVTREFNATDRAQQQKVCARYTTCEQMSLSNILEQERKRTEVSY
FG09254.1 	YFNDLYGYLETGPPVYFVTREVDASKRKEQQAICSRFTTCQDLSLPNTLELERQRPEVSY
AN2119.1  	YFDDLSEYFNSGPPVYFVTRNVNITKREHQRQLCGRFTTCEEYSLPFVLEQESKRSNVSY
          	**:**  *:::********:: : :.* .*: :*.*:***:: *:.  ** * :*.::**

MG00819.1 	IASPTASWIDDFFLWLDPDLGESCCVENGKACFADRNPPWSITMSGMPKGQEFVHYLDKF
NCU04090.1	ISTPTASWIDDFFQWLNPEN-ERCCMERRRPCFANRTPAWNITLSGMPEGDEFVYYLKKF
FG09254.1 	IASPAASWIDDYFLWLNPIF-EDCCVEHGQTCFADRVPAWNTTLYGMPEDEEFIHYLKKF
AN2119.1  	IAGATASWIDDFFYWLNPQQ--DCCYEDGKLCFEGRTPGWNISLTGMPEGAEFIHYLEKW
          	*: .:******:* **:*     ** *  : ** .* * *. :: ***:. **::**.*:

MG00819.1 	IQSPTTEECPLGGQAAYGDAVVIDHEKTTIGASHFRTMHTPLRSQSDFIKAYASARRIAN
NCU04090.1	LSAPTNEDCPLGGQASYGSAVLVDSDRDTIPASHFRTSHIPLRSQEDFIDAYAAARRIAN
FG09254.1 	LSSPTGEECPLAGQAAYGQAVVLDSKENHIKSTHFRTMHSPLRSQEDFIAAYSAARRIAS
AN2119.1  	IKSPTDASCPLGGKAPYSNALVFDPKRITTNASHFRTSHTPLRTQDDFIKSYISARRIAD
          	:.:**  .***.*:*.*..*::.* ..    ::**** * ***:*.*** :* :*****.

MG00819.1 	DISAQTGTEVFPYSVFYIFFDQYASIVDLTLALVGSALALIFVISTVLLGSFITAAVVTV
NCU04090.1	EISAETGLDVFPYSVFYVFFDQYASIVSLTGALLGSAIGIIFIVASVLLGSLVTAAVVSF
FG09254.1 	DIGERTGVDVFPYSVFYIFFDQYLSIVPLTAGLLSAAVGIIFVVATVLLGSALTALVVSV
AN2119.1  	GLSAEHGIDVFPYSKTYIFFDQYVSIVQVAGTLLGSAVAIIFAITSILLGSVATGAVVTA
          	 :. . * :*****  *:***** *** ::  *:.:*:.:** ::::****  *. **: 

MG00819.1 	TVMMAVVDIVGAMAVMGVSLNAVSLVNLIICVGIAVEFCVHIARAFMFPSRTFMERAKNR
NCU04090.1	TVVMAIVDIIGAMAVMGVSLNAVSLVNLIICVGIAVEFCAHIARAFMFPSRSCMERAKNR
FG09254.1 	TVVMSVVDIMGSMALFNVSLNAVSLVNLIICVGISVEFCAHIARAFMYPSRTVMEGNSNA
AN2119.1  	TVIMIVIDIIGSMAISGVSLNAVSLVNLVICVGIGVEFCAHIARAFMFPSRSIMEIVPSK
          	**:* ::**:*:**: .***********:*****.****.*******:***: **   . 

MG00819.1 	FRGRDARTWTALANVGGSVFSGITVTKLLGVTVLAFTRSKIFEIYYFRVWVALVIFAATH
NCU04090.1	FRGRDARAWTALSNVGGSVFSGITVTKLLGVFVLGFTRSKIFEIYYFRIWVALVIFAATH
FG09254.1 	FRGRDARAWTALVNVGGSVFSGITVTKLLGVSVLAFTRSKIFEIYYFRVWLSLVIFAALH
AN2119.1  	FRGKDARSWTALVNVGGSVFSGITVTKLLGVCVLAFTRSKIFEIYYFRVWLALIIFAATH
          	***:***:**** ****************** **.*************:*::*:**** *

MG00819.1 	ALIFLPVALSLLGGEG~YVDPESIGGLEEDLAARRYRATL-DHYSDSEDDE-
NCU04090.1	ALVFLPVALSLVGGEG~YVDSESDGGLEEDLASRRYRALVPDADSESEDEGF
FG09254.1 	ALVFLPVALSIAGGQG~YVDPESEGTAAQDLTDRRWRAIRVNDNSDSEDEY-
AN2119.1  	ALIFLPVALSYFGGDG1YADPEASGGLEENLASRSYRSLLVDDDYDSDEY--
          	**:*******  **:* *.*.*: *   ::*: * :*:   :   :*::
```
